# Chemoprevention of cancer: current evidence and future prospects

**DOI:** 10.12688/f1000research.6684.1

**Published:** 2015-09-28

**Authors:** Vassiliki Benetou, Areti Lagiou, Pagona Lagiou

**Affiliations:** 1 Department of Hygiene, Epidemiology and Medical Statistics, University of Athens Medical School, Athens, GR-115 27, Greece; 2 Department of Public Health and Community Health, Faculty of Health Professions, Athens Technological Educational Institute (TEI Athens), Athens, Greece; 3 Department of Epidemiology, Harvard School of Public Health, Boston, MA, USA

**Keywords:** Cancer, chemoprevention, aspirin, nutrients, supplements, antiestrogens, antiandrogens, SERMs, aromatase inhibitors, vaccines, HBV, HPV

## Abstract

Cancer chemoprevention refers to the use of agents for the inhibition, delay, or reversal of carcinogenesis before invasion. In the present review, agents examined in the context of cancer chemoprevention are classified in four major categories—hormonal, medications, diet-related agents, and vaccines—and the main representatives of each category are presented. Although there are serious constraints in the documentation of effectiveness of chemopreventive agents, mainly stemming from the long latency of the condition they are addressing and the frequent lack of intermediate biomarkers, there is little disagreement about the role of aspirin, whereas a diet rich in vegetables and fruits appears to convey more protection than individual micronutrients. Among categories of cancer chemopreventive agents, hormonal ones and vaccines might hold more promise for the future. Also, the identification of individuals who would benefit most from chemopreventive interventions on the basis of their genetic profiles could open new prospects for cancer chemoprevention.

## Introduction

Cancer is a leading cause of death worldwide, ranking second in economically developed countries ^1, 2^. With several forms of cancer being poorly controlled through treatments, which themselves have serious side effects, and with the unavoidable limitations of cancer screening programs, chemoprevention and its potential have generated much hope and interest during the last decades. Cancer chemoprevention is the inhibition or reversal of carcinogenesis (before invasion) by intervention with pharmacologically active agents ^3^. The concept was introduced by Sporn and colleagues in the mid-1970s ^4^.

More than a decade ago, one of us (PL) contributed to an effort to summarize available options for chemoprevention and its potential ^5^. In the present article, we examine the current evidence on chemopreventive agents and whether there has been any progress in terms of discovery or use of these agents in the context of cancer prevention.

## Cancer chemopreventive agents

Cancer chemopreventive agents can be classified in four major categories: hormonal, medications, diet-related agents, and vaccines.

## A. Hormonal chemopreventive agents

All hormonal chemopreventive agents are relevant to steroid-related cancers. They can be classified in two subcategories: antiestrogens and antiandrogens.

### a. Antiestrogens

***i. Selective estrogen receptor modulators***. Selective estrogen receptor modulators (SERMs) form a diverse group of compounds that exhibit a varying level of tissue-specific estrogen receptor (ER) activity that can be antagonistic but also agonistic depending on the target tissue ^6, 7^. More specifically, SERMs exert an antagonistic activity on breast tissue and an agonist activity on skeletal system. Some SERMs have been reported to demonstrate an ER agonistic effect on the vagina and an antagonistic effect on the endometrium, the latter when combined with estrogen.

During the last decade, strong evidence on the effectiveness of SERMs for breast cancer prevention has accumulated ^8^. A meta-analysis combining data from nine clinical trials and comparing use of SERMs (tamoxifen, raloxifene, arzoxifene, or lasofoxifene) with placebo reported a significant decrease in breast cancer incidence with treatment compounds, both during treatment and for at least 5 years after completion ^9^. The reduction in risk was confined to ER-positive invasive breast cancer (for all SERMs used) and ductal carcinoma *in situ* (for all, except raloxifene). Strengthening the evidence from the abovementioned meta-analysis, a recently updated analysis from the International Breast Cancer Intervention Study I (IBIS-I) provided evidence for a long-term protective effect of tamoxifen after its cessation, for at least 20 years after use ^10^.

Risks associated with tamoxifen are increased occurrence of thromboembolic events, endometrial cancer, and all-cause mortality ^8, 9, 11^. Raloxifene has been associated mainly with an increase in thromboembolic events. The risk-to-benefit ratio of treatment with tamoxifen or raloxifene depends on age, race, breast cancer risk, and history of hysterectomy. Over the course of a 5-year period, postmenopausal women with an intact uterus have been reported to have a better risk-to-benefit ratio for raloxifene compared with tamoxifen, whereas for postmenopausal women without a uterus the risk-to-benefit ratio was similar for the two compounds ^8, 12^. Notwithstanding the reported results on cancer incidence, overall breast cancer mortality has not been shown to decrease in chemoprevention trials with SERMs conducted so far, and consequently some are questioning their role in reducing the overall burden of breast cancer ^13^.

***ii. Aromatase inhibitors***. Aromatase inhibitors (AIs) inhibit the enzyme aromatase, which catalyzes the aromatization procedure that converts androgens into estrogens. Recent data suggest that anastrozole and exemestane are both associated with reduced breast cancer incidence among women at increased risk for the disease ^8, 14– 16^. They are well tolerated, although some researchers point out that careful monitoring of adverse effects related to joint pain and menopausal symptoms should be implemented in large clinical trials ^17, 18^. AIs can be used as an alternative chemoprevention agent for high-risk postmenopausal women who desire chemoprevention and have contraindications for SERM use ^14, 19^.

Regarding recommendations for the use of antiestrogens in breast cancer prevention, the American Society of Clinical Oncology practice guidelines indicate the administration of tamoxifen for 5 years in women 35 years and older at increased risk of breast cancer in order to reduce the risk of ER-positive breast cancer. In postmenopausal women, 5-year regimens with raloxifene or exemestane should also be discussed as options ^19^. In the UK, the National Institute for Health and Care Excellence also provides detailed guidance on the use of tamoxifen or raloxifene for pre- or postmenopausal women who are at risk of familial breast cancer but who are not at increased risk of thromboembolic disease or endometrial cancer ^20^.

Despite recommendations, the use of SERMs as primary prevention drugs for breast cancer in clinical practice is considered limited ^19– 21^. Several reasons have been invoked, including fear of adverse effects, the lack of reasonably accurate and feasible methods for assessing individual risk, the lack of a marker to monitor cancer risk reduction, insufficient public and professional information, and medication costs ^8^.

### b. Antiandrogens

Both testosterone and dihydrotestosterone (DHT) are essential for normal growth and functioning of the prostate. The role of antiandrogens in prostate cancer prevention relies on the hypothesis that androgens may be implicated in the etiology of prostate cancer and that suppressing DHT synthesis may inhibit carcinogenesis ^22^. 5-alpha-reductase is the enzyme that converts testosterone to the more active intracellular androgen DHT; the antiandrogens 5-alpha-reductase inhibitors (5-ARIs) block the process by inhibiting this enzyme.

Two 5-ARIs, finasteride and dutasteride, have been tested as chemopreventive agents for prostate cancer. Two large randomized placebo-controlled trials, the Prostate Cancer Prevention Trial with finasteride and the Reduction by Dutasteride of Prostate Cancer Events (REDUCE), have reported a decreased incidence of low-grade prostate cancer; in both, however, an absolute increase in high-grade prostate cancer has also been observed ^23– 25^.

A meta-analysis of randomized clinical trials reported that 5-ARIs reduce the risk of prostate cancer among men who are screened regularly by using prostate-specific antigen (PSA) level ^26^. The beneficial effects, however, were confined to men with PSA levels of less than 4.0 ng/mL. Evidence was insufficient with respect to the optimal age to initiate treatment or duration of chemoprevention. Uncertainty was also expressed with respect to the impact of 5-ARIs on tumors with the greatest lethal potential, including those with Gleason scores of 8 to 10. In 2010, the US Food and Drug Administration (FDA) evaluated the results of trials and supported the conclusion drawn by the Oncologic Drugs Advisory Committee that finasteride and dutasteride do not have a favorable risk-to-benefit profile in order to be proposed for chemoprevention of prostate cancer among healthy men ^25^.

## B. Medications

In the past, only aspirin and other anti-inflammatory drugs would have been classified under this category of chemopreventive agents. More recently, however, interest has emerged about a potential cancer chemopreventive role of statins and metformin.

### a. Aspirin and other anti-inflammatory drugs

Inflammation is linked to carcinogenesis and hence it is reasonable to assume that agents with anti-inflammatory effects, like the non-steroidal anti-inflammatory drugs (NSAIDs), could have cancer chemopreventive properties. The main representative of NSAIDs is aspirin, but other compounds like indomethacin and piroxicam are included in this class of medications. Various hypotheses have been invoked to explain the chemopreventive properties of NSAIDs ^5, 27^. Most prominent among them is the hypothesis about cyclooxygenase (COX) inhibition. COX-1 and -2 are enzymes necessary for the synthesis of inflammatory prostaglandins from arachidonic acid, and NSAIDs inhibit these enzymes. COX-2 is believed to be overexpressed in the early stages of colon carcinogenesis. Selective COX-2 inhibitors have also been developed.

A large body of evidence, from both randomized trials and observational epidemiological studies, has strengthened the hypothesis that regular prophylactic aspirin use reduces incidence of and mortality from colorectal cancer in the general population. A favorable effect of aspirin has also been reported with respect to recurrence of adenomatous polyps as well as polyp load in individuals with hereditary colon cancer ^27– 31^. Although data are less extensive, studies have shown reductions in incidence of and mortality from esophageal, stomach, and other gastrointestinal cancers as well as inverse, though small in magnitude, associations with breast, prostate, and lung cancers ^27^.

Issues that remain to be clarified are the optimal dose and duration of use and appropriate ages for use in average-risk individuals. Reduced incidence and mortality have been seen for all daily doses of above 75 mg, but there is no clear indication of a greater reduction with increasing dose ^32^. In a recent systematic review, the authors concluded that prophylactic aspirin use for at least 5 years at daily doses ranging from 75 to 325 mg, starting between ages 50 and 65, has a favorable risk-to-benefit profile for cancer prevention in the average-risk general population in the developed world for both sexes. Larger benefits were observed for 10-year use, whereas longer use still seems beneficial ^33^.

Nevertheless, benefits need to be balanced against harms. The side effects of aspirin and NSAIDs, attributed to inhibition of COX-1 activity in platelets, include gastrointestinal track bleeding and intracranial or extracranial hemorrhage, but serious incidents are not common at ages of less than 70 years ^27, 34^. Overall, it seems important for evidence-based recommendations regarding the use of aspirin in chemoprevention to be integrated with those for cardiovascular disease prevention.

### b. Statins

Statins (3-hydroxy-3-methylglutaryl coenzyme A reductase inhibitors) are used as cholesterol-lowering drugs but have also drawn attention as potential cancer chemopreventive agents ^35– 37^. Reduction of mevalonate synthesis by inhibiting 3-hydroxy-3-methylglutaryl coenzyme A reductase has been invoked as a possible mechanism for a statin-induced suppression of tumor growth, induction of apoptosis, and inhibition of angiogenesis ^38, 39^.

In a meta-analysis of 40 studies, a modest decrease in colorectal cancer risk with statin use was observed, which, however, was statistically significant among observational studies but not among randomized controlled trials ^40^. Reports for reduction of risk with respect to other cancer sites, such as prostate and gastric cancer as well as esophageal cancer (especially adenocarcinoma among patients with Barrett’s esophagus), have also appeared in the literature ^37, 41, 42^.

Overall, however, there is currently no conclusive evidence for a cancer chemopreventive effect of statins ^37, 43^. Of note, statin use among cancer patients before diagnosis has been associated with reduced total and site-specific mortality ^44^.

### c. Metformin

Metformin is a commonly prescribed drug for type 2 diabetes and belongs in the biguanide class. The crucial role of energy metabolism in cell growth and proliferation implies that antidiabetic or metabolism-altering drugs may hold preventive and therapeutic value, and, in this context, mechanisms for a potential cancer preventive effect of metformin have been proposed ^45^.

Epidemiologic studies indicate that diabetics treated with metformin have a decreased cancer risk compared with those on other antidiabetic medications ^46, 47^. Although evidence of a cancer chemopreventive effect of metformin among diabetics is accumulating, the question remains as to whether metformin can exert similar beneficial effects in non-diabetics ^45, 48, 49^.

## C. Diet-related agents

Several micronutrients have attracted the attention of the scientific community as potential cancer-preventive agents. Among them, diet-derived antioxidants have been studied intensively on account of the protection they convey against oxidative stress. Current evidence on chemopreventive effects of antioxidants and other micronutrients is summarized below.

### a. Carotenoids

Carotenoids are fat-soluble red/orange pigments with antioxidant properties and comprise more than 600 compounds. Of the approximately 50 found in human diets, only about half can be absorbed. Carotenoids are found in vegetables and include xanthophylls (e.g., lutein) and carotenes (e.g., beta-carotene and lycopene). Beta-carotene and other carotenoids can be converted to retinol and therefore are referred to by some as “pro-vitamin A” ^50^.

Beta-carotene is one of the most studied carotenoids. Observational epidemiologic studies have shown a beneficial effect of beta-carotene dietary intake on cancer prevention, but large clinical trials conducted during the 1990s did not confirm these findings; on the contrary, they demonstrated a detrimental effect. Thus, in the Alpha-Tocopherol Beta-Carotene (ATBC) Cancer Prevention Study, beta-carotene supplementation was associated with an increase in lung cancer risk as well as in risk of other cancers, notably prostate and stomach ^51^. Similarly, in the Beta-Carotene and Retinol Efficacy Trial (CARET), beta-carotene and retinol supplementation were found to increase lung cancer risk ^52^. Overall, the evidence about a protective effect of foods rich in carotenoids against cancers of the mouth, lung, pharynx, and larynx as well as about a protective effect of foods rich in beta-carotene against esophageal cancer is evaluated as probable; there is, however, strong evidence that beta-carotene supplements are associated with lung cancer in current smokers ^50, 53^, which has been classified as “convincing” in the second expert report of the World Cancer Research Fund and the American Institute for Cancer Research ^50^. Among other carotenoids, dietary lycopene (mainly found in tomatoes) has been inversely associated with prostate cancer risk, but the level of evidence has been downgraded from probable ^50^ to limited ^54^.

### b. Vitamin A and retinoids

Vitamin A or retinol is the best known retinoid. Retinoids are required for the maintenance of normal cell growth and differentiation; together with their dietary precursor (beta-carotene), they were some of the first agents to be tested in large population-based trials ^55^. The CARET trial in the United States studied beta-carotene along with retinol among smokers and did not show benefit from retinol (nor for beta-carotene) supplementation ^52^. Both the ATBC and the CARET trials found a significant increase in lung cancer incidence in the retinol/beta-carotene-containing arms ^56^.

### c. Folic acid

Folic acid or folate, a water-soluble vitamin B, is an important cofactor in one-carbon metabolism. Folate appears to possess dual modulatory effects on colorectal carcinogenesis depending on timing and dosage. In normal colorectal mucosa folate deficiency appears to enhance neoplastic transformation, modest levels of folic acid supplementation appear to suppress, whereas high supplemental doses appear to enhance the development of cancer. Of note, folate deficiency appears also to inhibit whereas folate supplementation has a promoting effect on the progression of established colorectal neoplasms ^57^. On the basis of a lack of compelling supportive evidence from studies in humans and its potential tumor-promoting effect, folic acid supplementation cannot currently be recommended for colorectal cancer chemoprevention. The evidence concerning the inverse association of dietary folate intake with pancreatic cancer has been downgraded from probable ^50^ to limited ^58^.

### d. Vitamin C

Vitamin C is a water-soluble antioxidant and enzyme cofactor. Humans do not have the ability to synthesize it and must obtain it through diet. Vitamin C has two chemical forms, one reduced (ascorbic acid) and one oxidized (dehydroascorbic acid) form.

In 1997, expert panels at the World Cancer Research Fund (WCRF) and the American Institute for Cancer Research had concluded that dietary vitamin C could reduce the risk of the stomach (probably) as well as mouth, pharynx, esophagus, lung, pancreas, and cervical cancers (possibly), but in their updated report in 2007, only the evidence with respect to esophageal cancer was considered probable and there was no evidence that vitamin C supplementation modifies the risk of cancer ^50, 59^. Recently, in a large-scale clinical trial in men, vitamin C supplementation had no immediate or long-term effects on the risk of prostate or other site-specific cancers or total cancer ^60^.

### e. Vitamin D

Vitamin D plays an important role in calcium metabolism but also exerts various other physiological functions. Experimental studies have shown that many cell types, including colorectal cells, express vitamin D receptors, and activation of these receptors by 1,25(OH) _2_D (1,25-dihydroxycholecalciferol or calcitriol) has been reported to exert antitumor effects ^61^.

In 2008, the International Agency for Research on Cancer (IARC) Working Group on Vitamin D, having examined the evidence on various cancer sites, concluded that evidence from observational studies for an inverse association between serum 25-hydroxyvitamin D levels and the incidence of colorectal cancer and sporadic colorectal adenomas was consistent and persuasive, but there was only limited evidence of a causal association and this was due to possible confounding by other dietary or lifestyle factors. Results from randomized trials to that date had not demonstrated an effect of vitamin D supplementation on colorectal cancer risk but could not be judged as contradictory to the evidence from observational studies, either ^62^. Hence, there have been suggestions for a minimum vitamin D intake in the context of colorectal cancer prevention ^61^.

### f. Vitamin E

Vitamin E is a fat-soluble vitamin with antioxidant activity. It refers to a group of compounds that include both tocotrienols and tocopherols, among which α-tocopherol is the most biologically active.

In the ATBC trial, an inverse association between vitamin E supplementation and prostate cancer was reported but it disappeared post-interventionally ^63, 64^. Null results for vitamin E supplementation were also reported in the Physicians’ Health Study with respect to prostate as well as overall cancer ^60^. In the Selenium and Vitamin E Cancer Prevention Trial (SELECT) in men, vitamin E, alone or in combination with selenium, was not associated with a reduction in prostate cancer risk; a subsequent report even noted an increase in the risk of prostate cancer among those who received vitamin E ^65, 66^. Hence, current evidence does not support the use of vitamin E for cancer prevention ^67^.

### g. Calcium

Calcium is an essential nutrient and plays an important role in muscular contraction, cellular growth, cell adhesion, and bone formation. Results from large observational studies support a relatively consistent inverse association of calcium intake with colorectal adenomas and colorectal cancer ^61, 68^. This effect is thought to be exerted by binding to toxic secondary bile acids and ionized fatty acids to form insoluble soaps in the lumen of the colon or by directly reducing proliferation, stimulating differentiation, and inducing apoptosis in the colonic mucosa ^69^.

In a systematic review and meta-analysis, supplemental calcium was reported to be effective for the prevention of adenoma recurrence in populations with a history of adenomas, but no association was found for colorectal cancer ^70^.

There is some concern that a high calcium intake may increase prostate cancer incidence. The evidence on diets high in calcium or dairy products is considered as limited but suggestive, whereas the evidence on calcium supplements has been downgraded from probable to limited, on the basis of which no conclusions can be drawn ^50, 54^.

### h. Selenium

Selenium is an essential cofactor for the major antioxidant enzyme glutathione peroxidase, which protects against oxidative damage to lipids, lipoproteins, and DNA ^50, 71^. The results of the SELECT clinical trial did not provide encouraging data for prostate cancer prevention ^65^. Also, a more recent phase III study of selenium versus placebo in patients with high-grade prostatic intraepithelial neoplasia found no benefit in the prevention of progression to prostate cancer and even suggested that higher intake might increase the risk of cancer ^72^. In its updated report of 2014 on prostate cancer, the WCRF concluded that there is limited suggestive evidence that low plasma concentrations of selenium are associated with increased prostate cancer risk, but no conclusions can be drawn on the basis of the existing evidence for selenium supplementation ^54^.

### i. Flavonoids

Flavonoids are polyphenolic compounds that inhibit carcinogen-activating enzymes and possess various antioxidant properties. More than 5,000 individual flavonoids have been identified and have been classified into subclasses on the basis of their range and structural complexity. Fruits and vegetables, along with tea and wine, are the main dietary sources of flavonoids ^73^.

Epidemiologic data, though not conclusive, suggest a protective role of flavonoids on particular cancer types, such as lung, breast, colon, and prostate ^73– 78^. In a meta-analysis of observational studies, flavonol and flavone intake, but not other flavonoid subclass or total flavonoid intake, were associated with a decreased breast cancer risk, especially among post-menopausal women ^79^. With respect to colorectal cancer, evidence on the role of flavonoid intake was judged to be insufficient and conflicting ^80^. Evidence on a potential chemopreventive role of various polyphenols, such as isoflavones, on prostate cancer risk has also been reported ^81– 83^.

### j. Multivitamin/multimineral supplements

In 2007, the National Institutes of Health, in a State-of-the-Science statement on multivitamin/multimineral supplements and chronic disease prevention, indicated that data are scarce on the efficacy and safety of multivitamin and mineral supplement use in primary prevention of chronic diseases in the general adult population. Specifically for cancer, though the statement recognized a potential benefit among persons with poor nutritional status or suboptimal antioxidant intake, it concluded that use of multivitamin supplements by the general population was not supported by the existing scientific evidence ^84^. In 2013, a meta-analysis of randomized controlled trials concluded that multivitamin/multimineral use had no effect on cancer prevention ^85^. Of note, the expert panels of the WCRF report concluded that the evidence from its review of trials did not show that micronutrient supplements have any benefits in cancer survivors, whereas high-dose supplements may even be harmful ^50^.

## D. Vaccines for cancer prevention

Several infections have been linked to increased cancer risk; however, only two vaccines against infectious agents are currently used in clinical practice for the prevention of cancer: the vaccine against the hepatitis B virus (HBV) and the vaccine against human papilloma virus (HPV) ^86, 87^.

The HBV vaccine was developed in the late 1960s. The first commercial vaccine was circulated in the early 1980s; genetically engineered vaccines were developed in the late 1980s and these vaccines are the ones currently used. Chronic HBV infection is a major cause of hepatocellular carcinoma, and by preventing the infection and chronic carriage state, the HBV vaccine provides protection against hepatocellular carcinoma ^88^.

HPV vaccination was introduced much later than HBV vaccination. The first HPV vaccine was approved by the FDA in the mid-2000s. There are more than 40 HPV types that infect human mucosal surfaces, but most infections are asymptomatic and transient. However, certain oncogenic types that persist can cause cervical cancer and other, less common, cancers, including cancers of the anus, penis, vulva, vagina, and oropharynx. Other, non-oncogenic HPV types can cause genital warts ^89, 90^. Two preventive HPV vaccines, one quadrivalent (which protects against types 16, 18, 6, and 11) and one bivalent (which protects against types 16 and 18), are currently used, but research for new vaccines that will protect against more oncogenic types of the virus is ongoing ^91^. Although history of use of this vaccine is not long, current evidence suggests that it is both effective and safe ^91, 92^.

## Conclusions

The concept of chemoprevention is intuitively attractive as it implies avoidance of suffering caused by the diagnosis of cancer, the disease itself, and its treatment. There are, however, serious constraints in the documentation of effectiveness of chemopreventive agents for cancer ^11, 95^, mainly stemming from the long latency of the condition they are addressing. Cancer latency is in the range of years and is certainly longer than the duration of the clinical trials designed to address the effectiveness of cancer chemopreventive agents. The problem could be bypassed with the use of intermediate biomarkers, but these are frequently lacking. Of note, trials assessing the effectiveness of chemopreventive agents have to rely on changes in the incidence of cancer in a population, and this is a rare event, even among high-risk population groups. Other limitations in the documentation of effectiveness of chemopreventive agents are related to the preservation of bioactivity of the various compounds following digestion as well as to the determination of the appropriate effective dose. Furthermore, because chemoprevention refers to the widespread and long-term use of compounds by the general “healthy” population, safety is an issue of paramount importance that needs to be addressed in studies with long follow-up in large segments of the population in order to be able to identify even rare side effects.

Although the opinions about the potential of cancer chemoprevention vary widely in the literature, there is little disagreement about the role of aspirin, whereas a diet rich in vegetables and fruits appears to convey more protection than individual micronutrients ^11, 93– 95^. Among categories of chemopreventive agents, hormonal ones and vaccines used for cancer prevention might hold more promise for the future. Also, the potential of differential effectiveness of chemopreventive agents by particular genotypes ^96^ is extremely intriguing and could open new prospects for cancer chemoprevention.
