# Supplementary material for: Chemoprevention of cancer: current evidence and future prospects
Source: F1000Res. 2015 Sep 28;4(F1000 Faculty Rev):916. [Version 1] doi: 10.12688/f1000research.6684.1 (PMC4797967; doi:10.12688/f1000research.6684.1)
Supplement: Peer review history

# Peer review: Referee response for version 1

**DOI:** 10.5256/f1000research.7180.r10575

**Published:** 2015-09-28

**Authors:** Janusz Jankowski

**Affiliations:** 1 University Hospitals Coventry and Warwickshire, University of Warwick, Warwickshire, UK

I have read this submission. I believe that I have an appropriate level of expertise to confirm that it is of an acceptable scientific standard.

# Peer review: Referee response for version 1

**DOI:** 10.5256/f1000research.7180.r10574

**Published:** 2015-09-28

**Authors:** Hasan Mukhtar

**Affiliations:** 1 School of Medicine and Public Health, Department of Dermatology, University of Wisconsin Medical Science Center, Madison, WI, USA

I have read this submission. I believe that I have an appropriate level of expertise to confirm that it is of an acceptable scientific standard.

# Peer review: Referee response for version 1

**DOI:** 10.5256/f1000research.7180.r10573

**Published:** 2015-09-28

**Authors:** Michihiro Mutoh

**Affiliations:** 1 Epidemiology and Prevention Division, Research Center for Cancer Prevention and Screening, National Cancer Center, Chuo-ku, Japan

I have read this submission. I believe that I have an appropriate level of expertise to confirm that it is of an acceptable scientific standard.
